# Insight into the Taxonomic Resolution of the Pleosporalean Species Associated with Dead Woody Litter in Natural Forests from Yunnan, China

**DOI:** 10.3390/jof8040375

**Published:** 2022-04-06

**Authors:** Dhanushka N. Wanasinghe, Guang-Cong Ren, Jian-Chu Xu, Ratchadawan Cheewangkoon, Peter E. Mortimer

**Affiliations:** 1 Center for Mountain Futures, Kunming Institute of Botany, Chinese Academy of Sciences, Honghe 654400, China; wanasinghe@mail.kib.ac.cn (D.N.W.); J.C.Xu@cgiar.org (J.-C.X.); 2 Department of Entomology and Plant Pathology, Faculty of Agriculture, Chiang Mai University, Chiang Mai 50200, Thailand; 3 Center of Excellence in Fungal Research, Mae Fah Luang University, Chiang Rai 57100, Thailand; guangcong.ren@gmail.com; 4 Yunnan Key Laboratory for Wild Plant Resources, Department of Economic Plants and Biotechnology, Kunming Institute of Botany, Chinese Academy of Sciences, Kunming 650201, China; 5 CIFOR-ICRAF China Program, World Agroforestry (ICRAF), Kunming 650201, China

**Keywords:** *Ascomycota*, anamorph, *Dothideomycetes*, Greater Mekong Subregion, teleomorph

## Abstract

In the course of investigating the systematics of woody litter micromycete associates in Yunnan Province, China, we found one new species in *Phaeoseptaceae*, one new genus and three new species in *Sulcatisporaceae* from 16 specimens collected (ten collections of ascomycetous teleomorphs, four collections of hyphomycetous and two collections of coelomycetes anamorphs) from Ailaoshan, Chuxiong, Diqing, Honghe, Kunming, Lancang, Mengla and Yuxi in Yunnan Province. These taxonomic novelties were recognized with the aid of morphological comparisons and phylogenetic analyses of multiple gene sequences (non-translated loci and protein-coding regions). *Pleopunctum menglaense* sp. nov. is accommodated in *Phaeoseptaceae* (*Pleosporales*) based on its hyphomycetous anamorph, which is characterized by superficial sporodochia on the host surface, macronematous, mononematous, cylindrical, unbranched, aseptate, hyaline and smooth-walled conidiophores, monoblastic, terminal, hyaline conidiogenous cells, hyaline, muriform α conidia, and brown, muriform β conidia with tri-lobed wing like basal cells. *Kazuakitanaka* gen. nov. (type: *K*. *yuxiensis*) is introduced in *Sulcatisporaceae* (*Massarineae*, *Pleosporales*) for a saprobic ascomycete with teleomorphic and anamorphic (coelomycetous) features. The teleomorph possesses globose to subglobose ascomata with acentric ostiole, a peridial wall of textura angularis to textura prismatica, cylindric-clavate, pedicellate asci with an ocular chamber, and 1–2-septate, hyaline, fusiform, guttulate ascospores with a distinct mucilaginous sheath. The anamorph features pycnidial conidiomata, phialidic, ampulliform to cylindrical, hyaline conidiogenous cells and ampulliform to cylindrical, one-to-three-septate, hyaline, guttulate conidia. *Loculosulcatispora* was known only from its anamorph of *L*. *thailandica*. We observed the teleomorph of *Loculosulcatispora hongheensis* sp. nov. and amended the generic description of *Loculosulcatispora* accordingly. *Loculosulcatispora hongheensis* is characterized by globose to subglobose ascomata with a central ostiole, a peridial wall of textura angularis to globosa, branched, septate, pseudoparaphyses, clavate asci with a short pedicel and a minute ocular chamber and hyaline, fusiform, 1-septate ascospores with a thick irregular mucilaginous sheath. This study provides some insights into the diversity of fungi on dead woody litter in terrestrial habitats.

## 1. Introduction

Despite estimates that ~2.2–3.8 million or more fungal species exist on Earth [1], we know of only 146,150 [2], suggesting that 96% of fungal species remain unknown [3]. Some fungal groups are well researched because of their impact on human lives, while others remain seriously neglected. This could potentially distort our understanding of fungal diversity [4]. Owing to their abundance across ecosystems, the importance of fungi cannot be discounted in any region [5]. There are numerous understudied habitats that harbor abundant species, and if they were to be thoroughly studied, many new species could be found [6]. Most species of plant-associated fungi can be pathogens, endophytes, saprobes or epiphytes on a wide range of hosts that reside in terrestrial as well as aquatic habitats [7]. Particularly, microfungi play important functional roles in almost all ecosystems, functioning as decomposers that degrade dead organic materials and recycle nutrients for reuse in the ecosystem [8,9]. Given the omnipresent nature of microfungi, additional taxonomic and ecological knowledge is prerequisites to understanding microfungal biology and their environmental significance.

*Ascomycota* is the most species-rich phylum of *Fungi*, comprising more than 92,700 species [2], and *Dothideomycetes* is the largest and most ecologically diverse class of the phylum [10]. This class comprises saprobes, human and plant pathogens, endophytes, epiphytes, ectomycorrhizal, lichens, lichenicolous, nematode-trapping and rock-inhabiting members [11]. In the *Dothideomycetes*, *Pleosporales* is the most species-rich order, consisting of 10,142 species [2] recognized in more than 90 families and 650 genera [12]. In recent years, molecular studies coupled with morphological evidence have revealed numerous novel families, genera and species within *Pleosporales* [12]. Recently, many new woody litter pleosporalean lineages from terrestrial [5,11,13,14,15,16,17,18,19] or freshwater [20,21,22,23,24,25] environments have been reported in Yunnan Province, China.

At the Center for Mountain Futures (Kunming Institute of Botany), we are investigating the diversity of microfungi on woody litter [3,15,16,17,26,27]. During this survey of lignicolous ascomycetes in Yunnan, we encountered numerous undescribed pleosporalean fungi. This study aimed to document taxonomic novelties in *Phaeoseptaceae* and *Sulcatisporaceae*. We used sequence data from relevant members of *Pleosporales*, three functional ribosomal RNA genes, the small and large subunit of the nuclear ribosomal RNA (nc18S and nc28S), the internal transcribed spacer region (ITS) and two protein-coding genes, the second largest subunit of RNA polymerase II (*rpb*2) and translation elongation factor 1-alpha gene (*tef*1-α) to perform an in-depth phylogenetic study. We present morphological and molecular phylogenetic evidence that supports the recognition of a new species in *Phaeoseptaceae* and four new species in *Sulcatisporaceae*, including a new genus.

## 2. Materials and Methods

### 2.1. Isolates and Specimens

During our fieldwork across the Ailaoshan, Chuxiong, Diqing, Honghe, Kunming, Lancang, Mengla and Yuxi regions of Yunnan Province, China, typical black ascomata/conidiomata appearing on dead twigs were collected during both dry (January, March and April) and wet (May, June and July) seasons. In total, sixteen specimens were included in this study. The collected samples were placed in Ziploc bags and taken to the mycology laboratory of the Kunming Institute of Botany and stored inside paper envelopes. Single spore isolation was conducted, and germinated spores were handled in accordance with the methods described in Wanasinghe et al. [15]. Dried specimens (at room temperature) were preserved in the fungarium of the Cryptogams Kunming Institute of Botany, Academia Sinica (KUN-HKAS). Representative cultures were deposited in the Kunming Institute of Botany Culture Collection (KUMCC), Kunming, China and China General Microbiological Culture Collection Center (CGMCC). Nomenclatural data of fungal novelties were deposited in MycoBank [28].

### 2.2. DNA Extraction, PCR Amplifications and Sequencing

The extraction of genomic DNA was performed using fresh mycelia in accordance with the methods of Wanasinghe et al. [5], using the Biospin Fungus Genomic DNA Extraction Kit-BSC14S1 (BioFlux, Shanghai, China) and following manufacturer guidelines. When cultures could not be maintained for certain collected samples, DNA was extracted directly from the fruiting bodies of the fungus as outlined by Wanasinghe et al. [29]. The reference DNA for the polymerase chain reaction (PCR) was stored at 4 °C for regular use and stored at −20 °C for long-term storage.

Primers ITS5/ITS4 [30], LR0R/LR5 [31,32], NS1/NS4 [30], EF1-983F/EF1-2218R [33,34] and fRPB2-5f/fRPB2-7cR [35] were used to amplify the DNA sequences of the internal transcribed spacers (ITS), partial 28S large subunit rDNA (LSU), partial 18S small subunit rDNA (SSU), translation elongation factor 1-α (tef1), and RNA polymerase II second largest subunit (*rpb*2). Protocols used for PCR amplification (SSU, LSU, ITS and *tef*1) were used as in Wanasinghe et al. [15]. PCR amplification conditions of *rpb*2 were set as initial denaturation at 98 °C for 2 min, followed by 35 cycles of denaturation at 98 °C for 10 s, annealing at 52 °C for 10 s and extension at 72 °C for 20 s, with a final extension step at 72 °C for 5 min. The amplified PCR fragments were then sent to a private company for sequencing (BGI, Ltd., Shenzhen, China).

### 2.3. Molecular Phylogenetic Analyses

#### 2.3.1. Sequencing and Sequence Alignment

**Figure 1:**
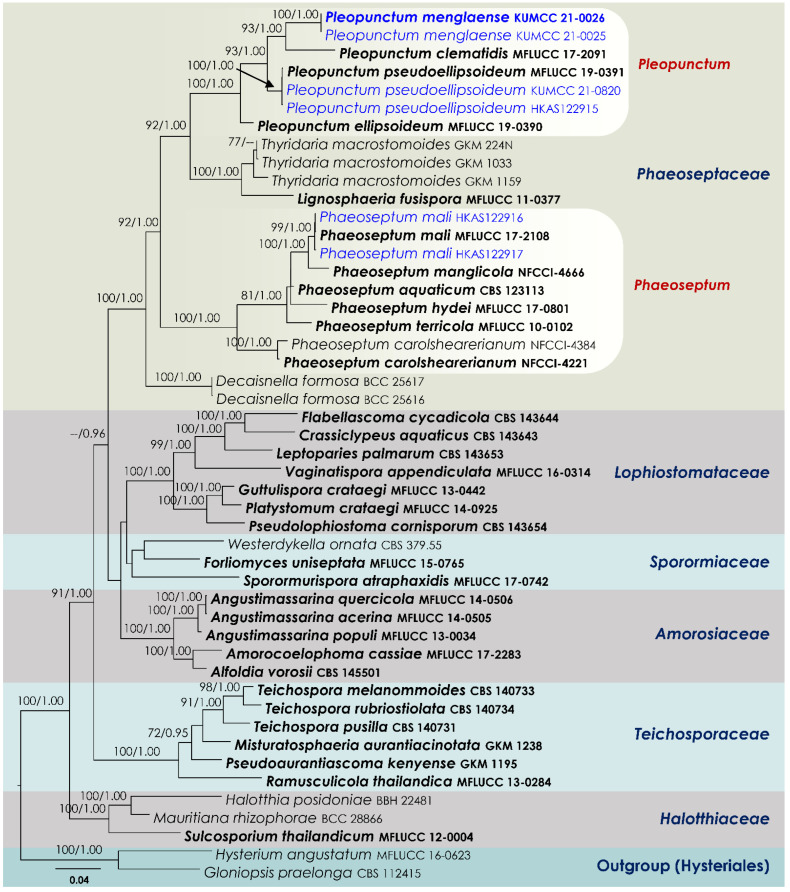
RAxML tree based on a combined dataset of a partial SSU, LSU, ITS, *tef*1 and *rpb*2 DNA sequence analysis in *Phaeoseptaceae*. Bootstrap support values for ML (MLB) equal to or greater than 70% and Bayesian posterior probabilities (BYPP) equal to or greater than 0.95 are shown as MLB/BYPP above the nodes. The new isolates are in blue. Species names given in bold indicate ex-type and ex-paratype strains. The scale bar represents the expected number of nucleotide substitutions per site.

**Figure 2:**
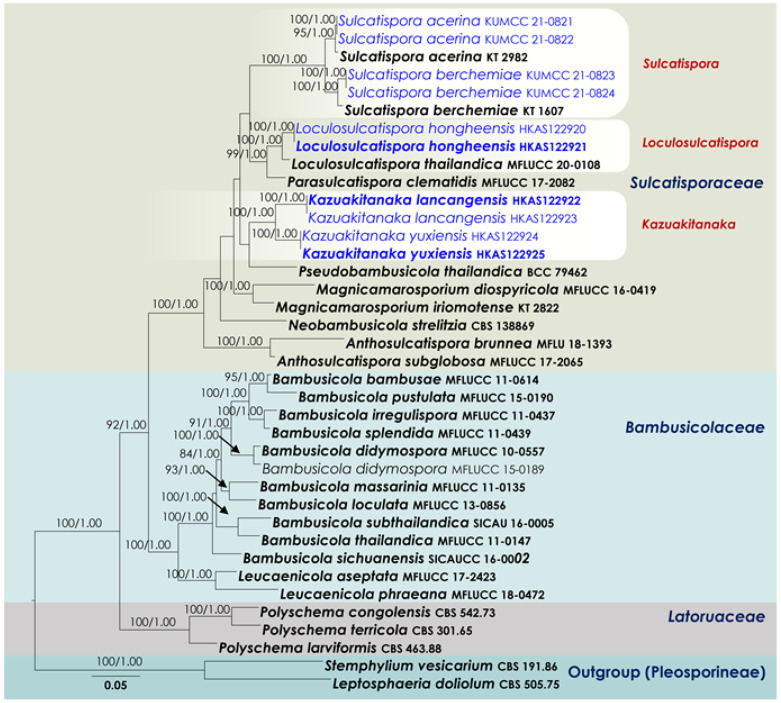
RAxML tree based on a combined dataset of a partial SSU, LSU, ITS, *tef*1 and *rpb*2 DNA sequence analysis in *Sulcatisporaceae*. Bootstrap support values for ML (MLB) equal to or greater than 70% and Bayesian posterior probabilities (BYPP) equal to or greater than 0.95 are shown as MLB/BYPP above the nodes. The new isolates are in blue. Species names given in bold indicate ex-type and ex-paratype strains. The scale bar represents the expected number of nucleotide substitutions per site.

BLAST searches using the BLASTn algorithm were performed to retrieve similar sequences from GenBank (http://www.ncbi.nlm.nih.gov, accessed on 29 December 2021) and relevant publications [36,37]. The collection/strain numbers for these sequences (Table 1) are presented in the corresponding phylogenetic trees (Figure 1 and Figure 2). All alignments were produced with the server version of MAFFT v.7 [38], checked and refined using BioEdit v.7.0.5.2 software [39].

#### 2.3.2. Phylogenetic Analyses

The single-gene datasets were examined for topological incongruence among loci and the conflict-free alignments were concatenated into a multi-locus alignment that was subjected to maximum-likelihood (ML) and Bayesian (BI) phylogenetic analyses. The evolutionary models for Bayesian analysis and maximum-likelihood were selected independently for each locus using MrModeltest v.2.3 [40] under the Akaike Information Criterion (AIC) implemented in PAUP v.4.0b10.

The CIPRES Science Gateway platform [41] was used to perform RAxML and Bayesian analyses. ML analyses were made with RAxML-HPC2 on XSEDE v.8.2.10 [42] using the GTR+GAMMA swap model with 1000 bootstrap repetitions.

MrBayes analyses were performed setting GTR+I+G for two million generations, sampling every 1000 generations, ending the run automatically when the standard deviation of split frequencies dropped below 0.01, with a burnin fraction of 0.25. ML bootstrap values (MLB) equal to or greater than 70% and posterior probability in Bayesian statistics (BYPP) greater than 0.95 are given above each node of every tree.

Phylograms were visualized with the FigTree v1.4.0 program [43] and reorganized in Microsoft PowerPoint (2007) and Adobe Illustrator^®^ CS5 (Version 15.0.0, Adobe^®^, San Jose, CA, USA). The finalized alignments and trees were deposited in TreeBASE, submission ID:29570 (http://purl.org/phylo/treebase/phylows/study/TB2:S29570, accessed on 19 March 2022).

### 2.4. Morphological Observations

Ascomata, conidiophores and conidia from the natural substrates were rehydrated with tap water and examined with a Motic SMZ 168 series stereo-microscope (Motic Asia, Kowloon, Hong Kong). Morphological characteristics were examined via hand sectioning of sporocarps placed on water-mounted glass slides. The following characteristics were evaluated: ascomata/conidiomata diameter, height, color and shape; width of peridium; and height and diameter of ostioles. Microscopic photography was conducted using a Nikon ECLIPSE Ni (Nikon Instruments Inc., Melville, NY, USA) compound microscope with differential interference contrast (DIC) and phase contrast (PC) illumination. Images of microscopic structures were captured with a Canon EOS 600D (Canon Inc., Ōta, Tokyo, Japan) camera. Macroscopic images of colonies were documented using an iPhone XS Max (Apple Inc., Cupertino, CA, USA) with daylight. Measurements were made with the Tarosoft (R) Image Frame Work program and images used for figures were processed with Adobe Photoshop CS6 (Adobe Systems, San Jose, CA, USA).

## 3. Results

### 3.1. Phylogenetic Analyses

The final concatenated SSU–LSU–ITS–*tef*1–*rpb*2 alignment (Figure 1) of *Phaeoseptaceae* comprised 48 strains, including the outgroup taxa *Gloniopsis praelonga* (CBS 112415) and *Hysterium angustatum* (MFLUCC 16-0623). The final alignment contained a total of 4478 characters used for the phylogenetic analyses, including alignment gaps (available in TreeBASE). The RAxML analysis of the combined dataset yielded a best scoring tree with a final ML optimization likelihood value of −30565.564394. The matrix had 1930 distinct alignment patterns, with 31.15% undetermined characters or gaps. Parameters for the GTR + I + G model of the combined amplicons were as follows: estimated base frequencies, A = 0.242181, C = 0.257106, G = 0.272525, T = 0.228188; substitution rates, AC = 1.247807, AG = 3.156565, AT = 1.478256, CG = 1.137163, CT = 7.093906, GT = 1.000; proportion of invariable sites, I = 0.397831; gamma distribution shape parameter, α = 0.56366. Bayesian analyses generated 3201 trees (average standard deviation of split frequencies: 0.009446) from which 2401 were sampled after 25% of the trees were discarded as burn-in. The alignment contained a total of 1932 unique site patterns.

The family *Phaeoseptaceae* (SSU, LSU, ITS, *tef*1 and *rpb*2 phylogeny) was resolved into four distinct clades with 100 MLB and 1.00 BYPP support values. Four strains of *Pleopunctum* (KUMCC 21-0820, KUMCC 21-0025, KUMCC 21-0026 and HKAS122915) isolated in the study formed a well-supported clade with *P*. *clematidis* (MFLUCC 17-2091), *P*. *ellipsoideum* (MFLUCC 19-0390) and *P*. *pseudoellipsoideum* (MFLUCC 19-0391). Three strains of *Thyridaria macrostomoides* (GKM 224N, GKM 1033, GKM 1159) constituted a strongly supported monophyly with the ex-type strain of *Lignosphaeria fusispora* (MFLUCC 11-0377). Two of our new strains, HKAS122916 and HKAS122917, nested with the ex-type strain of *Phaeoseptum mali* MFLUCC 17-2108 with 100 MLB and 1.00 BYPP statistical support. Strains of *Phaeoseptum aquaticum*, *P*. *carolshearerianum*, *P*. *hydei*, *P*. *mali*, *P*. *manglicola* and *P*. *terricola* grouped as a monophyletic clade with strong support values in both maximum likelihood and Bayesian analyses. Finally, the two strains of *Decaisnella formosa* (BCC 25616, BCC 25617) formed a basal terminal clade in *Phaeoseptaceae* with 100 MLB and 1.00 BYPP support values.

The *Sulcatisporaceae* (SSU, LSU, ITS, *tef*1 and *rpb*2 phylogeny) alignment contained 38 isolates (Figure 2), and the tree was rooted to *Leptosphaeria doliolum* (CBS 505.75) and *Stemphylium vesicarium* (CBS 191.86). The final alignment contained a total of 4931 characters used for the phylogenetic analyses, including alignment gaps (available in TreeBASE). The RAxML analysis of the combined dataset yielded a best scoring tree with a final ML optimization likelihood value of −27594.247903. The matrix had 1770 distinct alignment patterns, with 23.64% undetermined characters or gaps. Parameters for the GTR + I + G model of the combined amplicons were as follows: estimated base frequencies, A = 0.241395, C = 0.257298, G = 0.268694, T = 0.232613; substitution rates, AC = 1.256046, AG = 2.926191, AT = 1.154561, CG = 0.858049, CT = 6.686514, GT = 1.000; proportion of invariable sites, I = 0.499455; gamma distribution shape parameter, α = 0.532594. The Bayesian analyses generated 401 trees (average standard deviation of split frequencies: 0.009088) from which 301 were sampled after 25% of the trees were discarded as burn-in. The alignment contained a total of 1771 unique site patterns.

The family Sulcatisporaceae was composed of distinct lineages that correspond to the genera Anthosulcatispora, Loculosulcatispora, Magnicamarosporium, Neobambusicola, Parasulcatispora, Pseudobambusicola and Sulcatispora. Four strains (HKAS122922, HKAS122923, HKAS122924, HKAS122925) obtained in the present study formed a well-separated clade in Sulcatisporaceae, featuring both a single locus and concatenated datasets. Thus, this new lineage is presented here as the new genus Kazuakitanaka gen. nov. Two of our new isolates, KUMCC 21-0821 and KUMCC 21-0822, were grouped with the ex-type strain of Sulcatispora acerina (KT 2982) with 95 MLB and 1.00 BYPP values. Another two new strains (KUMCC 21-0823 and KUMCC 21-0824) grouped with the ex-type strain of Sulcatispora berchemiae (KT 1607), with 100 MLB and 1.00 BYPP support values. Loculosulcatispora thailandica (MFLUCC 20-0108), a type of Loculosulcatispora, constituted a monophyletic clade with two of our new isolates (HKAS122920 and HKAS122921). Neobambusicola strelitzia, Parasulcatispora clematidis and Pseudobambusicola thailandica were affiliated as monotypic genera. Two species of Magnicamarosporium, M. diospyricola (MFLUCC 16-0419) and M. iriomotense (KT 2822), grouped with 100 MLB and 1.00 BYPP support values. Anthosulcatispora brunnea (MFLU 18-1393) and A. subglobosa (MFLUCC 17-2065) formed a basal terminal clade in Sulcatisporaceae, with 100 MLB and 1.00 BYPP statistical support.

### 3.2. Taxonomy

***Pleosporales*** Luttr. ex M.E. Barr, Prodromus to class Loculoascomycetes: 67 (1987).

***Phaeoseptaceae*** Boonmee, Thambug. and K.D. Hyde, Mycosphere 9 (2): 323 (2018).

***Phaeoseptum*** Y. Zhang, J. Fourn. and K.D. Hyde, Mycologia 105 (3): 606 (2013).

***Phaeoseptum mali*** Phukhams. and K.D. Hyde, Asian Journal of Mycology 2 (1): 120 (2019).

Material examined: China, Yunnan, Honghe Hani and Yi Autonomous Prefecture, Kaiyuan, 23.863601° N, 103.407975° E, 1053 m, on dead woody litter, 17 March 2019, D.N. Wanasinghe (HKAS122916), ibid. Chuxiong Yi Autonomous Prefecture, Shuangbai County, 24.80527° N, 101.933887° E, 1736 m, on dead woody litter under the *Fagaceae* species, 25 June 2019 (HKAS122917).

Notes: Phukhamsakda et al. [44] introduced *Phaeoseptum mali* from the bark of fallen twigs of *Malus halliana* from the botanical garden of the Kunming Institute of Botany, Yunnan, China. *Phaeoseptum mali* fits well within the generic descriptions of *Phaeoseptum* based on its globose and immersed ascomata, cellular pseudoparaphyses, cylindrical-clavate, pedicellate asci, allantoid and brown muriform ascospores. In this study, we collected another two specimens of *Phaeoseptum mali* from Chuxiong and Honghe Prefectures in Yunnan, China on dead woody litter. Multi-gene phylogenetic analyses of combined SSU, LSU, ITS, *tef*1 and *rpb*2 DNA sequence showed that the ex-type strain of *Phaeoseptum mali* (MFLUCC 17-2108) and our two strains (HKAS122916 and HKAS122917) are monophyletic with 100% MLB and 1.00 BYPP support values. Morphologically, these new collections are not different from the holotype of *Phaeoseptum mali*.

***Pleopunctum*** N.G. Liu, K.D. Hyde and J.K. Liu, Mycosphere 10 (1): 767 (2019).

**Figure 3:**
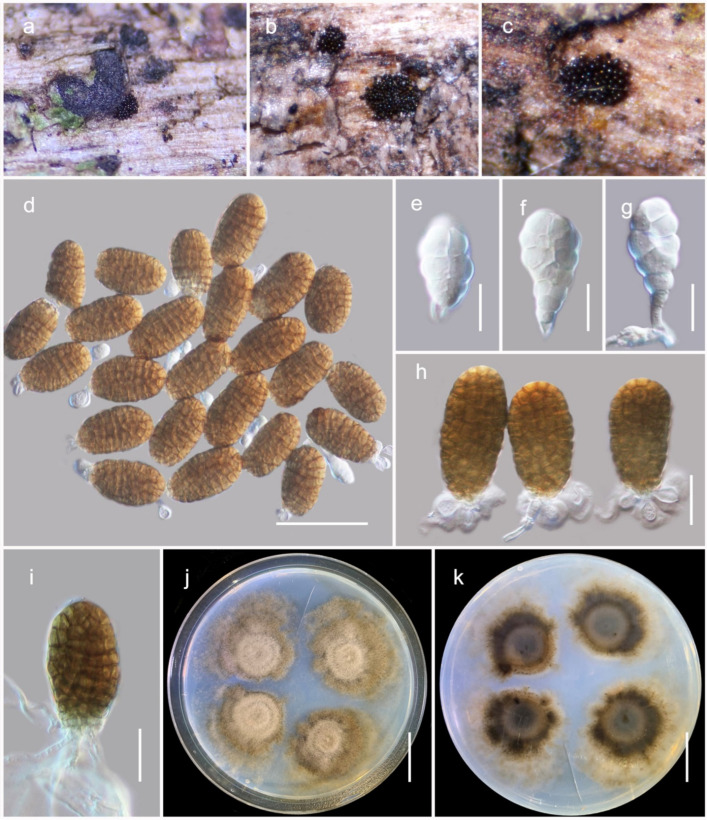
The anamorph of *Pleopunctum menglaense* (HKAS122683, holotype). (**a**–**c**) Colonies on host surface; (**d**) β conidia with basal cells; (**e**,**f**) α conidia showing remnant of conidiogenous cells at base; (**g**) α conidia with the conidiophore;(**h**) β conidia with distinct basal cells; (**i**) germinating conidium; (**j**,**k**) colonies on PDA after 21 days. Scale bars: (**d**) 50 μm; (**e**–**g**) 10 μm; (**h**,**i**) 20 μm; (**j**,**k**) 2 cm.

***Pleopunctum menglaense*** Wanas. sp. nov. (Figure 3).

MycoBank: MB843430.

Etymology: The specific epithet is derived from Mengla County, Yunnan Province, China.

Holotype: HKAS122683.

The species is a saprobe on dead twigs of forest litter in terrestrial habitats. Teleomorph: undetermined. Anamorph: hyphomycetous. Colonies on host, sporodochial, superficial, black, scattered and punctiform. The conidiophores are arising from hyaline unbranched hyphae. They are 10–20 × 2.5–3.5 μm (M = 16.7 × 3.1, *n* = 15), macronematous, mononematous, cylindrical, unbranched, aseptate, hyaline and smooth-walled. The conidiogenous cells 5–8 × 2.8–3.5 µm (M = 6.9 × 3.2 μm, *n* = 30) are monoblastic, terminal, hyaline. The conidia are dimorphic, acrogenous and solitary. The α conidia are 18–25 × 10–14 µm (M = 23.1 × 11.7 μm, *n* = 30), hyaline, multi-septate, muriform, spatulate to obovate, notably constricted at septa, slightly obtuse to rounded at apex, notably narrow at base, often carrying remnants of conidiogenous cells at base. The β conidia are 38–55 × 20–26 µm (M =  45.6 × 24.4 μm, *n* = 30), brown, muriform, ellipsoidal to oblong shaped, moderately rough-walled, and slightly constricted at septa often with a hyaline, elliptical to globose, 1-3 basal cells, 7.5–12 × 4–6 µm (M = 9 × 5.1 μm, *n* = 25) with a tri-lobed wing-like appearance.

Culture characteristics: the conidia germinated on PDA within 12 h and germ tubes were produced from the basal cells of conidia. The colonies on PDA reached a 5 cm diameter after 2 weeks at 25 °C. They were effused, circular to lobed, with incised margin, flat or slightly hairy, pinkish brown in the center with concentric rings and pale brown at the periphery and reverse; three distinct color zones were present, namely greyish brown at the center, pinkish brown at the middle, golden brown at the periphery.

Material examined: China, Yunnan, Xishuangbanna Dai Autonomous Prefecture, Mengla County, Wangtianshu, 21.61852° N, 101.58171° E, 747 m, on dead woody litter, 12 January 2021, D.N. Wanasinghe (HKAS122683, holotype), ex-type living culture, KUMCC 21-0826. *ibid*. 21.618839° N, 101.581098° E, 774 m (HKAS122684), living culture, KUMCC 21-0827.

***Pleopunctum pseudoellipsoideum*** N.G. Liu, K.D. Hyde and J.K. Liu, Mycosphere 10 (1): 768 (2019)

Material examined: China, Yunnan, Diqing Tibetan Autonomous Prefecture, Weixi Lisu Autonomous County, Zili Village, 27.803791° N, 99.059136° E, 2250 m, on dead woody litter, 25 June 2019, L.Q. Xian (HKAS122914), living culture, KUMCC 21-0820, ibid. Kunming, Luquan Yi and Miao Autonomous County, 26.052826° N, 102.670548° E, 2629 m, on dead woody litter under the *Fagaceae* species, 2 July 2019, D.N. Wanasinghe (HKAS122915).

Notes: Liu et al. [45] introduced *Pleopunctum pseudoellipticum* from decaying wood in Guizhou Province, China as a hyphomycetous muriform spored anamorph taxon in *Phaeoseptaceae*. During our investigation on the diversity of woody litter microfungi in Yunnan, China, two collections were made from the Diqing and Kunming areas. Morphological characteristics of our new collections, such as conidiophores and conidia, fit well within the anamorph of *Pleopunctum pseudoellipticum*. In our phylogenetic study (Figure 1), the new strains (KUMCC 21-0820, HKAS122915) cluster with *Pleopunctum pseudoellipticum* (MFLUCC 19-0391, ex-type strain) as a monophyletic clade with 100% MLB and 1.00 BYPP support values. Comparison of ITS, LSU and *tef*1 sequence data reveals there is no significant difference between our new isolates and *Pleopunctum pseudoellipticum*. Therefore, we recognize our new isolates as additional collections of *Pleopunctum pseudoellipticum*, and we provide SSU and *rpb*2 sequence data that were not provided by Liu et al. [45].

***Sulcatisporaceae*** Kaz. Tanaka and K. Hiray., Studies in Mycology 82: 119 (2015).

***Kazuakitanaka*** Wanas. gen. nov.

MycoBank: MB843431.

Etymology: The generic epithet stems from the combined two words “kazuaki” and “tanaka”, referring to Kazuaki Tanaka, who introduced the family Sulcatisporaceae.

The genus comprises saprobic fungi on woody substrates in terrestrial habitats. Teleomorph: the ascomata is a solitary or gregarious, semi-immersed, erumpent through the host surface, coriaceous, dark brown to black, globose to subglobose, ostiolate. The ostiole is central, with hyaline to pale-brown pseudoparenchymatous cells. The peridium is broader at the apex and thinner at the base, comprising two strata with several layers of brown or lightly pigmented to hyaline cells building textura angularis to textura prismatica, indistinguishable from the host tissues. The hamathecium comprises many branched, septate, cellular pseudoparaphyses, located between and above the asci, embedded in a gelatinous matrix. The asci are eight-spored, bitunicate, fissitunicate, cylindric-clavate, pedicellate, and apically rounded, with an ocular chamber. The ascospores are uni- to bi-seriate, partially overlapping in lateral view, and are hyaline. They are fusiform, 1–2-septate, slightly curved, deeply constricted at the central septum, conically rounded at the ends, and smooth-walled, with a distinct mucilaginous sheath, comprising a large guttule in each cell. Anamorph: Coelomycetous. The conidiomata are pycnidial, solitary, gregarious, dark brown to black, immersed or slightly erumpent, coriaceous to carbonaceous, papillate or apapillate. The conidiomatal wall is multi-layered; the outer layers are pseudoparenchymatous, with brown-walled cells, with the innermost layer thin and hyaline. The conidiophores are reduced to conidiogenous cells. The conidiogenous cells are phialidic, ampulliform to cylindrical, determinate, hyaline, smooth-walled and formed from the inner layer of the pycnidium wall. The conidia are hyaline, one-to-three-septate, straight to curved, fusiform, with conical ends, thick-walled, smooth, with large guttules in each cell.

Type species: *Kazuakitanaka yuxiensis*

**Figure 4:**
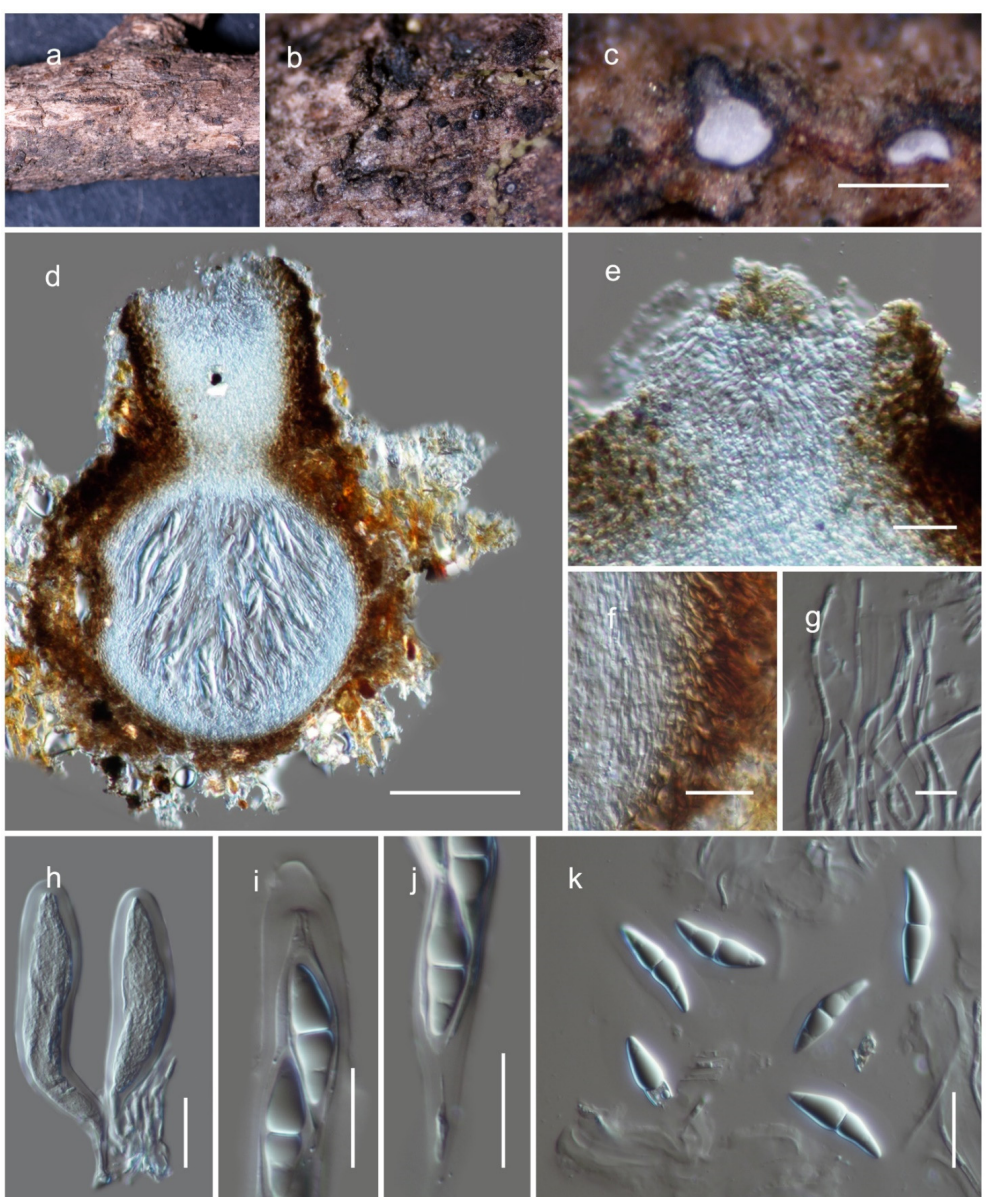
The teleomorph of *Kazuakitanaka yuxiensis* (HKAS122924, holotype). (**a**,**b**) Ascomata on dead woody twigs; (**c**,**d**) vertical section of ascomata; (**e**) closeup of ostiole; (**f**) peridium; (**g**) pseudoparaphyses; (**h**–**j**) asci; (**k**) ascospores. Scale bars: (**c**) 500 µm; (**d**) 100 µm; (**e**,**f**,**h**–**k**) 20 µm; (**g**) 10 µm.

***Kazuakitanaka yuxiensis*** Wanas. sp. nov. (Figure 4).

MycoBank: MB843432.

Etymology: The specific epithet is derived from Yuxi, Yunnan Province, China.

Holotype: HKAS122924.

The species is a saprobe on dead twigs of forest litter in terrestrial habitats. Teleomorph: the ascomata is 350–400 µm high, 220–260 µm diam. (M = 386.6 × 249.9 µm, *n* = 5), scattered to gregarious, semi-immersed to erumpent, coriaceous, dark brown to black, globose to subglobose ostiolate. The ostiole is 110–140 µm long, 90–115 µm diam. (M = 125.6 × 98.7 µm, *n* = 5), centrally papillate, comprising hyaline cells. The peridium is 10–15 µm wide at the base, 12–20 µm wide at the sides, broad at the apex (30–40 µm), comprising two strata, with the outer stratum composed of small, pale brown to brown, slightly flattened, thick-walled cells of textura angularis, fusing and indistinguishable from the host tissues. The inner stratum is composed of several layers with lightly pigmented to hyaline cells centrally textura angularis to textura prismatica. The hamathecium is 1–2 µm wide, branched, septate, cellular pseudoparaphyses, situated between and above the asci, embedded in a gelatinous matrix. The asci are 80–120 × 17–21 µm (M = 102.2 × 18.5 µm, *n* = 20), eight-spored, bitunicate, fissitunicate, cylindric-clavate, with a pedicel, and is rounded at the apex, with an ocular chamber. The ascospores are 25–32 × 7–9 µm (M = 29.1 × 7.9 µm, *n* = 30), uni- to bi-seriate, overlapping, and hyaline. They are fusiform, 1–2-septate. They are slightly curved, deeply constricted at the central septum, conically rounded at the ends, and smooth-walled, surrounded by a distinct mucilaginous sheath, each cell with a large guttule. Anamorph: undetermined.

Material examined: China, Yunnan, Yuxi, Xinping Yi and Dai Autonomous County, 24.09083° N, 101.935124° E, 2091 m, on dead woody litter, 25 May 2019, D.N. Wanasinghe (HKAS122924, holotype). ibid. 24.09083° N, 101.935124° E, 2095 m (HKAS122925).

**Figure 5:**
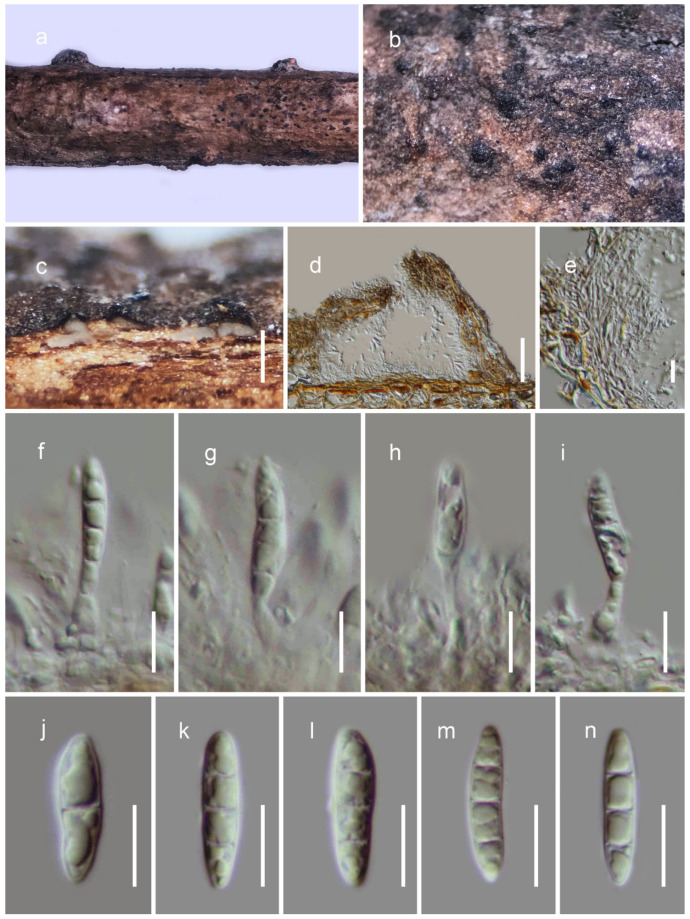
*Kazuakitanaka lancangensis* (HKAS122922, holotype). (**a**,**b**) Conidiomata on host surface; (**c**,**d**) sections through conidiomata; (**e**) conidioma wall; (**f**–**i**) conidiogenous cells and developing conidia; (**j**–**n**) conidia. Scale bars: (**c**) 500 µm; (**d**) 50 μm; (**e**) 15 μm; (**f**–**n**) 10 μm.

***Kazuakitanakalancangensis*** Wanas. sp. nov. (Figure 5).

MycoBank: MB843433.

Etymology: The specific epithet is derived from Lancang, Yunnan Province, China.

Holotype: HKAS122922.

It is saprobic on dead twigs of forest litter in terrestrial habitats. Teleomorph: undetermined. Anamorph: coelomycetous. The conidiomata is 130–150 µm high, 160–190 µm diam. (M = 140 × 170 µm, *n* = 5), pycnidial, solitary, gregarious, globose to subglobose, coriaceous, uni-loculate, dark brown to black, and immersed, with a central ostiole. The conidiomata wall is 10–20 µm wide, multi-layered, with brown-walled pseudoparenchymatous cells, with a hyaline inner most layer. The conidiophores are reduced to conidiogenous cells. The conidiogenous cells are 7–10 × 2–3.5 μm (M = 8.3 × 2.7 µm, *n* = 15), phialidic, ampulliform to cylindrical, determinate, hyaline, smooth-walled and formed from the inner layer of the pycnidium wall. The conidia are 17–23 × 3.7–4.7 2 µm (M = 20 × 4.2 µm, *n* = 30), fusiform, straight, hyaline, one-to-three-septate, not constricted at septa, tip and base rounded, thick-walled, and smooth, with numerous large guttules in each cell.

Material examined: China, Yunnan, Pure, Lancang Lahu Autonomous County, 23.164681° N, 99.969248° E, 1990 m, on dead woody litter, 11 April 2019, D.N. Wanasinghe (HKAS122922, holotype). ibid. 23.164686° N, 99.969492° E, 1975 m (HKAS122923).

***Loculosulcatispora*** G.C. Ren and K.D. Hyde, Phytotaxa 475 (2): 70 (2020) *amended*

MycoBank: MB557580

The genus comprises saprobic species on dead wood or twigs. Teleomorph: the ascomata are scattered, immersed to semi-immersed, globose to subglobose, brown to dark-brown, with a central ostiole. The peridium is composed of small, pale brown to brown, thick-walled cells forming textura angularis to *globosa*. The hamathecium comprises branched, septate, cellular pseudoparaphyses. The asci are eight-spored, bitunicate, clavate, straight to curved, with a short pedicel. They are apically rounded, with or without an ocular chamber. The ascospores are overlapping uni- to bi-seriate, hyaline, fusiform with acute ends, 1-septate, smooth-walled, surrounded by a thick irregular mucilaginous sheath. Anamorph: see Ren et al. [36].

Type species: *Loculosulcatispora thailandica* G.C. Ren and K.D. Hyde, Phytotaxa 475 (2): 73 (2020).

**Figure 6:**
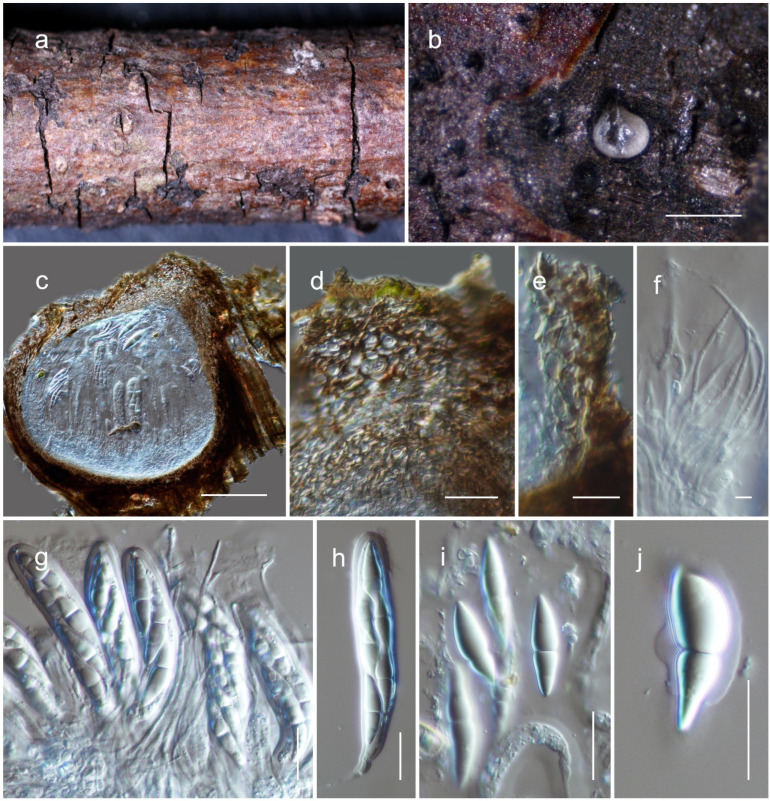
*Loculosulcatispora hongheensis* (HKAS122920, holotype). (**a**) Dead wood host substrate; (**b**,**c**) section of ascoma; (**d**); close-up of ostiole; (**e**) peridium; (**f**) pseudoparaphyses; (**g**,**h**) asci; (**i**,**j**) ascospores. Scale bars: (**b**) 500 µm; (**c**) 100 μm; (**d**,**g**,**h**) 20 μm; (**e**,**i**,**j**) 10 μm; (**f**) 5 μm.

***Loculosulcatispora hongheensis*** Wanas. sp. nov. (Figure 6).

MycoBank: MB843434.

Etymology: The specific epithet is derived from Honghe, Yunnan Province, China.

Holotype: HKAS122920.

The species is a saprobe on dead twigs of forest litter in terrestrial habitats. Teleomorph: the ascomata is 130–490 × 120–560 (M = 260 × 280, *n* = 5) μm, scattered, immersed, coriaceous, globose to subglobose, brown to dark-brown, with a central ostiole. The peridium is 10–15 μm wide; the outer stratum composed of small, pale brown to brown, thick-walled cells forming textura angularis to globosa indistinguishable from the host tissues. The inner stratum is composed of hyaline cells forming textura angularis. The hamathecium is 1–2.5 µm wide, branched, septate, cellular pseudoparaphyses, situated between and above the asci, embedded in a gelatinous matrix. The asci are 60–100 × 10–18 μm (M = 84 × 13.7 μm; *n* = 15) μm, eight-spored, bitunicate, fissitunicate, clavate, straight to curved, with a short pedicel. They are apically rounded, with a minute ocular chamber. The ascospores are 20–30 × 4–6.5 μm (M = 25 × 5 μm, *n* = 30), overlapping uni- to bi-seriate, hyaline, fusiform with acute ends, 1-septate, constricted at the septum, slightly longer upper cell than lower cell, slightly curved, smooth-walled, and surrounded by a thick irregular mucilaginous sheath. Anamorph: undetermined.

Material examined: China, Yunnan, Honghe Hani and Yi Autonomous Prefecture, Mengzi, 23.18604° N, 103.413838° E, 1854 m, on dead woody litter, 16 March 2019, D.N. Wanasinghe (HKAS122920, holotype). ibid. 23.186552° N, 103.414063° E, 1866 m (HKAS122921).

***Sulcatispora*** Kaz. Tanaka and K. Hiray., Studies in Mycology 82: 120 (2015).

***Sulcatispora acerina*** Kaz. Tanaka and K. Hiray., Studies in Mycology 82: 120 (2015).

Material examined: China, Yunnan, Pure, Jingdong Yi Autonomous County, 24.549205° N, 101.027521° E, 2560 m, on dead woody litter, 8 June 2021, D.N. Wanasinghe (HKAS122926), living culture, KUMCC 21-0821. ibid. 24.548691° N, 101.027802° E, 2546 m (HKAS122927), living culture, KUMCC 21-0822.

Notes: *Sulcatispora acerina*, introduced by Tanaka et al. [46], was collected from *Acer palmatum* in a terrestrial habitat. Based on phylogenetic analysis of combined SSU, LSU, ITS, *tef*1 and *rpb*2 sequence data, two of our isolates, KUMCC 21-0821 and KUMCC 21-0822, were clustered with the ex-type strain of *Sulcatispora acerina* (KT 2982) with 95% MLB and 1.00 BYPP support (Figure 2). Our isolate resembles *Sulcatispora acerina* in shape and size of the ascomata, asci and ascospores. Moreover, there are no base pair differences of the SSU and LSU nucleotides and only four and seven nucleotide differences in the ITS and *tef*1 regions, respectively. Therefore, we recognize that our new isolates belong to *Sulcatispora acerina* as new records from China, providing *rpb*2 sequence data that were not provided by Tanaka et al. [46].

***Sulcatispora berchemiae*** Kaz. Tanaka and K. Hiray., Studies in Mycology 82: 121 (2015).

Material examined: China, Yunnan, Pure, Jingdong Yi Autonomous County, 24.548237° N, 101.026484° E, 2516 m, on dead woody litter, 8 June 2021, D.N. Wanasinghe (HKAS122918), living culture, KUMCC 21-0823. ibid. 24.548142° N, 101.026572° E, 2510 m (HKAS122919), living culture, KUMCC 21-0824.

Notes: In the phylogenetic analysis (Figure 2), two of our new isolates (KUMCC 21-0823, KUMCC 21-0824) clustered with the type strain of *Sulcatispora berchemiae* (KT 1607) with high bootstrap support (100% MLB, 1.00 BYPP). These three strains share similar morphological features in ascomata, asci and ascospores. KT 1607 was collected from Japan on *Berchemia racemosa*, whereas KUMCC 21-0823 and KUMCC 21-0824 were collected from China on dead woody litter. Based on both morphology and molecular data, we consider our new isolates and *Sulcatispora berchemiae* to be conspecific. While extending the biogeography of *Sulcatispora berchemiae*, we also provide the *rpb*2 gene region for the species, which was not accounted for earlier.

## 4. Discussion

Hyde et al. [47] established *Phaeoseptaceae* in *Pleosporales* to accommodate *Phaeoseptum*. However, this family is now recognized as a group of heterogenous taxa that have diversified habitats with different types of anamorphs and diverged morphological characteristics [37]. In the recent outlines of Hongsanan et al. [10] and Wijayawardane et al. [12], only *Phaeoseptum* and *Pleopunctum* are accepted in *Phaeoseptaceae*. In the combined multi-gene phylogenetic analyses, the ex-type strains of *Lignosphaeria diospyrosa*, *L*. *fusispora* and *L*. *thailandica* also clustered in *Phaeoseptaceae* [48]. Therefore, *Lignosphaeria* should be an accepted genus within *Phaeoseptaceae*. Even though the putative strains of *Decaisnella formosa* (BCC 25616, BCC 25617) and *Thyridaria macrostomoides* (GKM 1033, GKM 1159, GKM 224N) are phylogenetically affiliated in *Phaeoseptaceae* [37,47], they are not related to any type specimens. Therefore, it is necessary to recollect and epitypify them with DNA sequence data in order to ensure correct generic placement of these two species [49,50].

*Phaeoseptum* was introduced by Zhang et al. [51] to accommodate *P*. *aquaticum*, and it was initially described as an accepted species in *Halotthiaceae* based on phylogenetic analysis of LSU sequence data. Currently, six species are accepted in *Phaeoseptum* viz.: *P*. *aquaticum*, *P*. *carolshearerianum*, *P*. *hydei*, *P*. *mali*, *P*. *manglicola* and *P*. *terricola* [10]. *Phaeoseptum aquaticum* was reported from freshwater habitats, and later Hyde et al. [47] added *P*. *terricola* from a terrestrial environment. In recent studies, Phukhamsakda et al. [44] and Wanasinghe et al. [37] collected *Phaeoseptum mali* and *P*. *hydei* from terrestrial habitats, respectively. Dayarathne et al. [52] showed that these species can be found in tropical coastlines, while introducing *Phaeoseptum carolshearerianum* and *P*. *manglicola* from mangroves. In this study, we accounted for another two records of *Phaeoseptum mali* from Chuxiong and Honghe Prefectures in Yunnan (China) from terrestrial habitats.

*Pleopunctum*, typified by *P*. *ellipsoideum*, is a hyphomycetous genus established in *Phaeoseptaceae* by Liu et al. [45]. Species in the genus are characterized by macronematous, mononematous conidiophores with monoblastic conidiogenous cells and oval to ellipsoidal, muriform conidia mostly with a hyaline, elliptical to globose basal cell [45]. Currently, five *Pleopunctum* species (*viz*. *P*. *bauhiniae*, *P*. *clematidis*, *P*. *ellipsoideum*, *P*. *pseudoellipsoideum*, *P*. *thailandicum*) are accepted in Species Fungorum [53]. However, Koukol and Delgado [54] argued that *Pleopunctum clematidis* should be a synonym of *P*. *bauhiniae* (=*Hermatomyces bauhiniae*). Members in the genus have appeared to be saprobic on dead twigs of deciduous hosts in terrestrial habitats [45,55,56,57]. In this study, morphological characteristics and multi-gene phylogenetic analysis of combined SSU, LSU, ITS, *tef*1 and *rpb*2 DNA sequence data reveals a new species of *Pleopunctum* from the dead woody litter collected in Yunnan, China. In a multi-gene (concatenated LSU, SSU, ITS, *tef*1 and *rpb*2) phylogenetic analysis, two of our strains (KUMCC 21-0025, KUMCC 21-0026) clustered with *Pleopunctum* species as a monophyletic clade with 100 MBP and 1.00 BYPP bootstrap support (Figure 1). Morphological features of KUMCC 21-0025 and KUMCC 21-0026 are similar to extant species of *Pleopunctum* in having sporodochial colonies, holoblastic, monoblastic conidiogenous cells and brown muriform conidia [45]. We introduce these two isolates as a novel species belonging to this genus, *Pleopunctum menglaense*. Within the *Pleopunctum* clade, *P. menglaense* strains constitute a sister lineage to *P*. *pseudoellipsoideum* (MFLUCC 19-0391, KUMCC 21-0820, HKAS122915). *Pleopunctum menglaense* has contrasting morphological features to *P*. *pseudoellipsoideum* by its distinct, hyaline, 1–3 basal cells in β conidia, whereas *P. pseudoellipsoideum* has a single basal cell in β conidia [45].

*Sulcatisporaceae* currently accommodates seven genera: viz. *Anthosulcatispora, Loculosulcatispora, Magnicamarosporium, Neobambusicola, Parasulcatispora, Pseudobambusicola* and *Sulcatispora* [12]. The species in this family are mostly restricted to terrestrial habitats excluding *Neobambusicola strelitziae*, which was found from the coastal region (Eastern Cape Province) of South Africa [58]. The conidia of anamorphs of *Sulcatisporaceae* can vary from hyaline, aseptate or septate (*Anthosulcatispora*, *Loculosulcatispora*, *Neobambusicola*, *Pseudobambusicola*), to pigmented phragmo-conidia (*Sulcatispora*) or muriform conidia (*Magnicamarosporium*), with or without striation [36,46,56,58,59,60]. The anamorph of *Parasulcatispora* is yet to be discovered. Shared characteristics within the family consist of immersed ascomata, peridium comprising pseudoparenchymatous cells with a thickened apex, cellular or trabeculate pseudoparaphyses and having cylindrical to cylindric-clavate asci with a long pedicel. However, the ascospore arrangement inside an ascus and the features of ascospores can differ between species belonging to this group. The spore arrangement can be uniseriate (*Anthosulcatispora*) and 2–3-seriate in others. Spores are hyaline and fusiform with conical ends that are completely surrounded by a sheath excluding *Anthosulcatispora brunnea*, which is brown to dark brown, oblong to ellipsoidal and lacking a sheath.

*Kazuakitanaka* is introduced in *Sulcatisporaceae* as a new genus, based on LSU, ITS, *tef*1 and *rpb*2 sequence data. *Kazuakitanaka* morphologically resembles *Parasulcatispora* and *Sulcatispora* with its cylindric-clavate asci and fusiform, 1-septate hyaline ascospores. However, these genera were revealed as phylogenetically distant in multi-gene phylogenetic analysis (Figure 2). The coelomycetous anamorph of *Kazuakitanaka* is similar to species of *Neobambusicola* and *Pseudobambusicola* by its pycnidial conidiomata, phialidic conidiogenous cells and hyaline, fusiform, multi-septate conidia. The new genus has a close phylogenetic proximity to *Pseudobambusicola* even though this relationship is not statistically significant, and *Neobambusicola* was placed in a distinct branch apart from the *Kazuakitanaka* clade (Figure 2). The teleomorph for these genera is still undetermined and therefore they cannot be morphologically compared with the teleomorph of the new genus.

Ren et al. [36] introduced *Loculosulcatispora* as a monotypic genus to accommodate *L*. *thailandica*, and it was known only from its anamorph. *Loculosulcatispora thailandica* was introduced as a decaying woody-based saprobe from Thailand (Chiang Rai). The genus was characterized by multilocular conidiomata, phialidic, discrete, determinate, doliiform to cylindrical, hyaline conidiogenous cells, unbranched and aseptate paraphyses, and 1-celled, oblong conidia with guttules. During our sample collections, we found a teleomorphic species characterized by globose to subglobose, immersed ascomata with a central ostiole, peridium composed of textura angularis, hamathecium comprises branched, septate, cellular pseudoparaphyses, clavate, short pedicellate asci with a minute ocular chamber, hyaline, fusiform, and 1-septate ascospores with a mucilaginous sheath. In the multi-gene phylogenetic analyses, two of the isolates of this teleomorphic species (HKAS122920, HKAS122921) constituted a monophyletic clade (100% MLB and 1.00 BYPP, Figure 2) with the type strain of *Loculosulcatispora thailandica* (MFLUCC 20-0108). Therefore, we introduce our new collections as *Loculosulcatispora hongheensis* sp. nov. and amended the generic descriptions herein to accommodate its teleomorphic characteristics. The *Loculosulcatispora* clade has a sister affiliation to *Parasulcatispora clematidis*, which is known only from its teleomorph [56]. It could also be a species in *Loculosulcatispora*. For us to combine these two genera here would require an extensive taxonomic reassessment and describing anamorphic morphology, which is beyond the scope of the current study..

Tanaka et al. [46] introduced *Sulcatispora* to accommodate *S*. *acerina* and *S*. *berchemiae*, which were collected as saprobes on *Sapindaceae* (*Acer palmatum*) and *Rhamnaceae* (*Berchemia racemosa*) hosts. The teleomorph of the genus is characterized by globose, subglobose to hemispherical and immersed to erumpent ascomata, short papillate, central ostioles with periphyses, peridium composed of several layers of compressed angular cells, trabeculate pseudoparaphyses, clavate asci with a short pedicel and fusiform, 1-septate, hyaline ascospores with a large sheath. The anamorph is characteristic with globose, pycnidial conidiomata, cylindrical and annellidic conidiogenous cells, ellipsoid, yellowish brown, multi-septate conidia with striate ornamentation. These teleomorphic characteristics are somewhat similar to those in *Massarina*. The most distinctive feature of *Sulcatispora* is the longitudinal striae on the surface of conidia. Some species in *Barriopsis* (e.g., *B*. *iraniana*), *Dwiroopa* (e.g., *D*. *ramya*), *Endomelanconium* (e.g., *E*. *pini*), *Lasiodiplodia* (e.g., *L*. *theobromae*), *Mucoharknessia* (e.g., *M*. *cortaderiae*), *Neodeightonia* (e.g., *N*. *phoenicum*) and *Phaeophleospora* (e.g., *P*. *striae*) are known to have such conidia [61].

In the present study, we identified five genera and eight species associated with dead woody litter in Yunnan, China. Among them, one genus and four species are new to science. Our results emphasize that Yunnan Province has not yet been properly studied and is an open field for new fungal discoveries.

## Figures and Tables

**Table 1:** Taxa used in the phylogenetic analyses and their corresponding GenBank numbers. Isolates/sequences in bold were isolated/sequenced in the present study.

Species	Strain ^1^	GenBank Accession Numbers				
		SSU	LSU	ITS	*tef*1	*rpb*2
*Alfoldia vorosii*	CBS 145501 ^T^	MK589346	MK589354	JN859336	MK599320	-
*Amorocoelophoma cassiae*	MFLUCC 17-2283 ^T^	NG_065775	NG_066307	NR_163330	MK360041	MK434894
*Angustimassarina acerina*	MFLUCC 14-0505 ^T^	NG_063573	KP888637	NR_138406	KR075168	-
*Angustimassarina populi*	MFLUCC 13-0034 ^T^	NG_061204	KP888642	KP899137	KR075164	-
*Angustimassarina quercicola*	MFLUCC 14-0506 ^T^	NG_063574	KP888638	KP899133	KR075169	-
*Anthosulcatispora brunnea*	MFLU 18-1393 ^T^	-	MH644791	MH644792	-	-
*Anthosulcatispora subglobosa*	MFLUCC 17-2065 ^T^	MT226705	MT214592	MT310636	MT394649	MT394706
*Bambusicola bambusae*	MFLUCC 11-0614 ^T^	JX442039	JX442035	JX442031	KP761722	KP761718
*Bambusicola didymospora*	MFLUCC 10-0557 ^T^	KU872110	KU863105	KU940116	KU940188	KU940163
*Bambusicola didymospora*	MFLUCC 15-0189	KU872111	KU863106	KU940117	KU940189	KU940164
*Bambusicola irregulispora*	MFLUCC 11-0437 ^T^	JX442040	JX442036	JX442032	KP761723	KP761719
*Bambusicola loculata*	MFLUCC 13-0856 ^T^	KP761735	KP761729	KP761732	KP761724	KP761715
*Bambusicola massarinia*	MFLUCC 11-0135 ^T^	KU872115	KU863111	KU940122	KU940192	KU940169
*Bambusicola pustulata*	MFLUCC 15-0190 ^T^	KU872112	KU863107	KU940118	KU940190	KU940165
*Bambusicola sichuanensis*	SICAUCC 16-0002 ^T^	MK253528	MK253532	MK253473	MK262828	MK262830
*Bambusicola splendida*	MFLUCC 11-0439 ^T^	JX442042	JX442038	JX442034	KP761726	KP761717
*Bambusicola subthailandica*	SICAU 16-0005 ^T^	MK253529	MK253533	MK253474	MK262829	MK262831
*Bambusicola thailandica*	MFLUCC 11-0147 ^T^	KU872113	KU863108	KU940119	KU940191	KU940166
*Crassiclypeus aquaticus*	CBS 143643 ^T^	LC312472	LC312530	LC312501	LC312559	LC312588
*Decaisnella formosa*	BCC 25616	GQ925833	GQ925846	-	GU479851	-
*Decaisnella formosa*	BCC 25617	GQ925834	GQ925847	-	GU479850	-
*Flabellascoma cycadicola*	CBS 143644 ^T^	LC312473	LC312531	LC312502	LC312560	LC312589
*Forliomyces uniseptata*	MFLUCC 15-0765 ^T^	NG_061234	NG_059659	NR_154006	KU727897	-
*Gloniopsis praelonga*	CBS 112415	FJ161134	FJ161173	-	FJ161090	FJ161113
*Guttulispora crataegi*	MFLUCC 13-0442 ^T^	KP899125	KP888639	KP899134	KR075161	-
*Halotthia posidoniae*	BBH 22481	GU479752	GU479786	-	-	-
*Hysterium angustatum*	MFLUCC 16-0623	GU397359	FJ161180	-	FJ161096	MH535875
***Kazuakitanaka lancangensis***	**HKAS122922 ^T^**	**ON009088**	**ON009104**	**ON009120**	**ON009263**	**-**
***Kazuakitanaka lancangensis***	**HKAS122923**	**ON009089**	**ON009105**	**ON009121**	**ON009264**	**-**
***Kazuakitanaka yuxiensis***	**HKAS122924**	**ON009092**	**ON009108**	**ON009124**	**ON009267**	**ON009290**
***Kazuakitanaka yuxiensis***	**HKAS122925 ^T^**	**ON009093**	**ON009109**	**ON009125**	**ON009268**	**ON009291**
*Leptoparies palmarum*	CBS 143653 ^T^	LC312485	LC312543	LC312514	LC312572	LC312601
*Leptosphaeria doliolum*	CBS 505.75 ^T^	GU296159	GU301827	NR_155309	GU349069	KY064035
*Leucaenicola aseptata*	MFLUCC 17-2423 ^T^	NG_065776	MK347963	NR_163332	MK360059	MK434891
*Leucaenicola phraeana*	MFLUCC 18-0472 ^T^	NG_065784	MK348003	MK347785	MK360060	MK434867
*Lignosphaeria fusispora*	MFLUCC 11-0377 ^T^	-	KP888646	NR_164233	-	-
*Loculosulcatispora thailandica*	MFLUCC 20-0108 ^T^	MT383968	MT383964	MT376742	MT380476	MT380475
***Loculosulcatispora hongheensis***	**HKAS122920**	**ON009090**	**ON009106**	**ON009122**	**ON009265**	**ON009288**
***Loculosulcatispora hongheensis***	**HKAS122921 ^T^**	**ON009091**	**ON009107**	**ON009123**	**ON009266**	**ON009289**
*Magnicamarosporium diospyricola*	MFLUCC 16-0419 ^T^	NG_065102	KY554212	NR_153486	KY554209	KY554208
*Magnicamarosporium iriomotense*	KT 2822 ^T^	AB797219	AB807509	AB809640	AB808485	-
*Mauritiana rhizophorae*	BCC 28866	GU371832	GU371824	-	GU371817	-
*Misturatosphaeria aurantiacinotata*	GKM 1238 ^T^	-	NG_059927	-	GU327761	-
*Neobambusicola strelitzia*	CBS 138869 ^T^	-	KP004495	NR_137945	MG976037	-
*Parasulcatispora clematidis*	MFLUCC 17-2082 ^T^	-	MT214593	MT310637	MT394650	-
*Phaeoseptum aquaticum*	CBS 123113 ^T^	-	JN644072	KY940803	-	-
*Phaeoseptum carolshearerianum*	NFCCI-4221 ^T^	MK307816	MK307813	MK307810	MK309874	MK309877
*Phaeoseptum carolshearerianum*	NFCCI-4384	MK307818	MK307815	MK307812	MK309876	MK309879
*Phaeoseptum hydei*	MFLUCC 17-0801 ^T^	MT240624	MT240623	MT240622	MT241506	-
***Phaeoseptum mali***	**HKAS122916**	**ON009082**	**ON009098**	**ON009114**	**ON009257**	**ON009282**
***Phaeoseptum mali***	**HKAS122917**	**ON009083**	**ON009099**	**ON009115**	**ON009258**	**ON009283**
*Phaeoseptum mali*	MFLUCC 17-2108 ^T^	-	MK625197	MK659580	MK647990	-
*Phaeoseptum manglicola*	NFCCI-4666 ^T^	MK307817	MK307814	MK307811	MK309875	MK309878
*Phaeoseptum terricola*	MFLUCC 10-0102 ^T^	MH105780	MH105779	MH105778	MH105781	-
*Pleopunctum ellipsoideum*	MFLUCC 19-0390 ^T^	MK804514	MK804517	MK804512	MK828510	-
***Pleopunctum pseudoellipsoideum***	**KUMCC 21-0820**	**ON009084**	**ON009100**	**ON009116**	**ON009259**	**ON009284**
***Pleopunctum pseudoellipsoideum***	**HKAS122915**	**ON009085**	**ON009101**	**ON009117**	**ON009260**	**ON009285**
*Pleopunctum pseudoellipsoideum*	MFLUCC 19-0391 ^T^	-	MK804518	MK804513	MK828511	-
*Pleopunctum thailandicum*	MFLUCC 21-0039 ^T^	-	MZ198896	MZ198894	MZ172461	-
***Pleopunctum menglaense***	**KUMCC 21-0025**	**ON009086**	**ON009102**	**ON009118**	**ON009261**	**ON009286**
***Pleopunctum menglaense***	**KUMCC 21-0026 ^T^**	**ON009087**	**ON009103**	**ON009119**	**ON009262**	**ON009287**
*Polyschema congolensis*	CBS 542.73 ^T^	-	EF204502	MH860770	-	EF204486
*Polyschema larviformis*	CBS 463.88 ^T^	-	EF204503	-	-	-
*Polyschema terricola*	CBS 301.65 ^T^	EF204519	EF204504	NR_160100	-	EF204487
*Pseudoaurantiascoma kenyense*	GKM 1195 ^T^	-	NG_059928	-	GU327767	-
*Pseudobambusicola thailandica*	BCC 79462	-	MG926560	MG926559	MG926562	MG926561
*Pseudolophiostoma cornisporum*	CBS 143654 ^T^	LC312486	LC312544	LC312515	LC312573	LC312602
*Ramusculicola thailandica*	MFLUCC 13-0284 ^T^	KP899131	KP888647	KP899141	KR075167	-
*Sporormurispora atraphaxidis*	MFLUCC 17-0742 ^T^	NG_061296	NG_059880	NR_157546	-	-
*Stemphylium vesicarium*	CBS 191.86 ^T^	DQ247812	DQ247804	MH861935	DQ471090	KC584471
***Sulcatispora acerina***	**KUMCC 21-0821**	**ON009096**	**ON009112**	**ON009128**	**ON009271**	**ON009294**
***Sulcatispora acerina***	**KUMCC 21-0822**	**ON009097**	**ON009113**	**ON009129**	**ON009272**	**ON009295**
*Sulcatispora acerina*	KT 2982 ^T^	LC014605	LC014610	LC014597	LC014615	-
*Sulcatispora berchemiae*	KT 1607 ^T^	AB797244	AB807534	AB809635	AB808509	-
***Sulcatispora berchemiae***	**KUMCC 21-0823**	**ON009094**	**ON009110**	**ON009126**	**ON009269**	**ON009292**
***Sulcatispora berchemiae***	**KUMCC 21-0824**	**ON009095**	**ON009111**	**ON009127**	**ON009270**	**ON009293**
*Sulcosporium thailandicum*	MFLUCC 12-0004 ^T^	KT426564	KT426563	MG520958	-	-
*Teichospora melanommoides*	CBS 140733 ^T^	-	KU601585	NR_154632	KU601610	-
*Teichospora pusilla*	CBS 140731 ^T^	-	KU601586	NR_154633	KU601605	-
*Teichospora rubriostiolata*	CBS 140734 ^T^	-	KU601590	NR_154634	KU601609	KU601599
*Thyridaria macrostomoides*	GKM 1033	-	GU385190	-	GU327776	-
*Thyridaria macrostomoides*	GKM 1159	-	GU385185	-	GU327778	-
*Thyridaria macrostomoides*	GKM 224N	-	GU385191	-	GU327777	-
*Vaginatispora appendiculata*	MFLUCC 16-0314 ^T^	KU743219	KU743218	KU743217	KU743220	-
*Westerdykella ornata*	CBS 379.55	GU296208	GU301880	AY943045	GU349021	-

^1^ Ex-type strains are mentioned with superscripted “T”.

## Data Availability

The datasets generated for this study can be found in the NCBI GenBank, MycoBank and TreeBASE.
